# Ascites in infantile onset type II Sialidosis

**DOI:** 10.1002/jmd2.12305

**Published:** 2022-06-03

**Authors:** Kaoutar Tazi, Vanessa Guy‐Viterbo, Alexander Gheldof, Aurélie Empain, Anne Paternoster, Corinne De Laet

**Affiliations:** ^1^ Paediatric Department Hôpital Universitaire des Enfants Reine Fabiola, Université Libre de Bruxelles Avenue Jean Joseph Crocq 15, 1020 Brussels Belgium; ^2^ Pediatric Intensive Care Unit Hôpital Universitaire des Enfants Reine Fabiola, Université Libre de Bruxelles Avenue Jean Joseph Crocq 15, 1020 Brussels Belgium; ^3^ Center for Medical Genetics Universitair Ziekenhuis Brussel, Vrije Universiteit Brussel Avenue du Laerbeek 101, 1090 Brussels Belgium; ^4^ Nutrition and Metabolic Unit Hôpital Universitaire des Enfants Reine Fabiola, Université Libre de Bruxelles Avenue Jean Joseph Crocq 15, 1020 Brussels Belgium; ^5^ Paediatric Department Centre Hospitalier EpiCURA Route de Mons 63, 7301 Hornu Belgium

**Keywords:** ascites, hypoalbuminemia, nephrosialidosis, portal hypertension, protein‐losing enteropathy, type II sialidosis

## Abstract

Sialidosis is a rare autosomal‐recessive lysosomal storage disease due to mutations in the *NEU1* gene leading to a deficit of alpha‐n‐acetyl neuraminidase and causing aberrant accumulation of sialylated glycoproteins/peptides and oligosaccharides in the lysosomes of various organs and tissues. Type II sialidosis (dysmorphic form) is classified into three subgroups based on the age of onset and the clinical severity: Congenital or neonatal, infantile (onset 0–12 months) and juvenile form (onset 13 months–20 years). We report the case of a 3‐year‐old boy with sialidosis type II infantile form, who developed a voluminous ascites. To the best of our knowledge, ascites is not described in the infantile form but in the congenital form of the disease. Ascites seems to be of a multifactorial origin regarding our investigations: on the one hand, portal hypertension and on the other hypoalbuminemia maintained by proteinuria secondary to nephrosialidosis. Loss of plasma proteins in the gastrointestinal tract (protein‐losing enteropathy) should also be considered in the case of portal hypertension and damages of the reticuloendothelial system.

**Synopsis**

The presence of ascites in the infantile form of sialidosis is likely of multifactorial origin and is probably suggestive of a rapidly advancing course of the disease associated with a poor prognosis.

## 1 INTRODUCTION

Sialidosis is a rare autosomal‐recessive lysosomal storage disease due to mutations in the *NEU1* gene, located in 6p21.3. *NEU1* encodes for the alpha‐n‐acetyl neuraminidase, a lysosomal enzyme that is part of a heterotrimeric complex together with beta‐galactosidase and cathepsin A. More than 60 *NEU1* mutations have been identified.^1^, ^2^ The resulting enzyme deficit leads to aberrant accumulation of sialylated peptides, glycoproteins and oligosaccharides in the lysosomes of various organs and tissues. The various *NEU1* mutations and the related residual neuraminidase activity are somewhat correlated with both the severity of the clinical manifestations and the onset age, demonstrating the existence of significant genotype–phenotype correlation in sialidosis.^4^, ^5^

The proper nosology of neuraminidase deficiency was given by Lowden and O'Brien (1979),^3^ who classified patients with sialidosis into two main types: Type I (normomorphic) and Type II (dysmorphic). Type I sialidosis is the non‐neuropathic form of the disease, also known as cherry‐red spot myoclonus syndrome. Symptoms appear in the second or third decade of life. The most prominent clinical manifestation is myoclonus with decreased visual acuity and bilateral macular cherry‐red spots. Patients have no obvious physical defects with normal or slightly impaired intelligence.^6^, ^7^ The clinical features of type II sialidosis are: typical storage disease features such as coarse face and hepatomegaly, developmental delay, dysostosis multiplex, myoclonus, macular cherry‐red spots, nystagmus, hearing loss, inguinal hernias, vertebral deformities. Renal impairment has been described but remains uncommon.^5^, ^6^, ^7^ Type II sialidosis is classified into three subgroups based on onset age and clinical severity: (A) Congenital or neonatal form with hydrops fetalis or ascites. Patients are either stillborn or die shortly after birth, (B) infantile form (0–12 months) and juvenile form (13 months–20 years).^5^, ^6^, ^7^ The treatment for sialidosis is supportive with no specific therapy currently available.^6^

We report the case of a 3‐year‐old boy with type II infantile sialidosis who developed a voluminous ascites of multifactorial origin.

## 2 CASE REPORT

The patient was born by spontaneous and uncomplicated vaginal delivery at 35 + 3/7 weeks gestation (Apgar 9/10/10). He was the third child of non‐consanguineous Belgian parents without any notable family history. His birth weight, height and head circumference were, respectively, 2310 kg (z‐score: −2.7), 45 cm (z‐score: −2.7) and 31 cm (z‐score: −2.9). The physical examination revealed congenital torticollis with an otherwise normal neurological examination. He necessitated 15 days in neonatal care for feeding difficulties. From the age of 4 months, he suffered from recurrent respiratory infections.

He was hospitalised at 8‐month‐old for failure to thrive (weight, *z*‐score − 4.1; height, z‐score: −4.5 and head circumference, z‐score: −1.2). On physical examination at admission, he was pale and hypotrophic with coarse facial features. Bilateral inguinal hernias and palpable hepatosplenomegaly were also noted. Neurological examination showed severe neurodevelopmental delay with axial hypotonia, poor ocular contact and loss of head carriage.

Laboratory tests revealed anaemia (Hb 8.2 g/dl), thrombocytopenia (platelets 73 000/μl), slightly elevated ASAT (58UI/L), hypoalbuminemia (29 g/L) and vitamin D deficiency (14.3 μg/L). Blood smear showed vacuolated lymphocytes. A bone marrow aspiration, revealed further vacuolated lymphocytes. The clinical and cytological findings were therefore suggestive of lysosomal storage disease. β‐Glucocerebrosidase activity measured on filter paper and serum oxysterol level were normal. A urine mucopolysaccharide profile was normal but the oligosaccharide profile was abnormal. Total sialic acid value was 10 371 μmol/g creatinine (six times the normal value) suggesting a diagnosis of either sialidosis or galactosialidosis. Gene panel sequencing for lysosomal storage diseases detected a heterozygous *NEU1* pathogenic mutation (NM_000434.3(NEU1):c.599C>T; p.(Pro200Leu), with a GnomAD allele frequency of 0.0009%, already described in patients with sialidosis,^8^ together with a *NEU1* heterozygous variant of uncertain clinical significance {(NM_000434.3(NEU1):c.353‐11A>G; p.?) with a GnomAD allele frequency of 0.004%} not described in the literature. However, the prediction tools SpliceSiteFinder, the two different mutations are on different alleles, as both parents are heterozygous for one mutation each. Enzymatic activity measurement of alpha neuraminidase in cultured skin fibroblasts was severely disturbed (0.12 nmol/mg/h; range normal activities 7–48 nmol/mg/h). At the same time, a control (enzymatic analysis on dermal fibroblasts of a person non affected with a Neu1 deficiency) was carried out by the laboratory. This confirmed the diagnosis of sialidosis.

A hearing test concluded to bilateral mixed hearing loss (70%). The heart ultrasound, brain MRI and ophthalmic assessment were normal. Abdominal ultrasound showed hepatosplenomegaly with homogeneous echo structure. There was no ascites, and the kidneys were normal. Broad metaphysis and enlargement of growth plates, suggesting rickets, were noted on skeletal X‐rays. The pulmonary investigations for recurrent respiratory infections showed a normal chest CT but bronchoscopy revealed left bronchomalacia. Neurological assessment reported severe developmental delay. Enteral feeding with concentrated milk was necessary to treat insufficient weight gain.

For the next 2 years follow‐up, he demonstrated developmental progress and was able to sit on his own, grab toys and say simple words. He developed nystagmus, dysostosis multiplex and dorsal kyphosis.

At the age of 3 years, he was hospitalised again because of voluminous ascites. The parents reported recent weight gain, bloating and abdominal discomfort. He was pale, uncomfortable and tachypnoeic with shallow breathing. The height, weight and head circumference were 9490 kg (z‐score: −3.4), 72 cm (z‐score: −6.4) and 48.5 cm (z‐score: −1), respectively. His frail limbs contrasted with his large abdomen (mid‐upper arm circumference of 10.5 cm = Z‐score < −3). He had a bloated, distended, shiny and painful abdomen with venous collateral circulation, telangiectasias and stretch marks. The hepatosplenomegaly had worsened since the last hospitalisation (+2 cm at midclavicular line). He had bilateral hydrocele and oedema of the lower limbs. He developed nail clubbing and generalised petechiae. Besides axial hypotonia, we noticed corneal haze and gingival enlargement. He presented with high blood pressure (122/66 mmHg) and hypoxemia (SpO2 90%).

Laboratory tests showed thrombocytopenia (Platelets 16 000/μl), anaemia (Hb 9.9 g/dl), hypoalbuminemia (27 g/L), hypertriglyceridemia (403 mg/dl), a mildly decreased glomerular filtration rate (GFR 80.3 ml/min/1.73 m^2^) and a low proteinemia of 52 g/L with a normal PT, aPTT and factor V measurement. Ascites aetiology was looked for by an extensive assessment.

A 24‐hour urine collection showed an increased proteinuria of 0.63 g per day and a protein/creatinine ratio of 7,3 g/g creatinine. Unfortunately, the abnormally low urinary creatinine suggests these results might be overestimated. The kidney ultrasound showed normal kidneys without either focal lesions or dilation of the pyelocaliceal cavities.

The abdominal ultrasound showed significant ascites. Abdominal paracentesis was carried out, showing abundant citrine ascitic fluid with a serum‐ascites albumin gradient greater than 1.1 g/dl suggesting non‐infected cheliform transudate in favour of portal hypertension. Echocardiography excluded post hepatic portal hypertension from congestive heart disease. Hepatic and Doppler ultrasound showed homogeneous echo structure without sign of Budd–Chiari syndrome, prehepatic obstruction, portal hypertension, fibrosis, cirrhosis or dilation of the bile ducts. Inferior vena cava, hepatic, portal and splenic veins were all patent. Doppler showed normal flow in the portal vein.

As the previous examinations were noncontributive, magnetic resonance angiography of the liver was performed. There were no lymphadenopathy, no mural thickening of the intestine, no thrombosis or compression syndrome; furthermore, splenic varices were identified. The massive hepatosplenomegaly (15.4 × 9.3 × 7.5 cm and 12.2 × 5.8 × 7.4 cm, respectively) was of homogeneous structure without associated damage.

An esophagogastroduodenoscopy was carried out to look for varices. Small oesophageal varices and micro‐petechiae on the gastric mucosa were seen, revealing portal hypertensive gastropathy.

Non‐cirrhotic portal hypertension was suspected (which concerns intrahepatic vascular involvement). An invasive measurement of portal pressure would have been the gold standard diagnostic test for precise identification of non‐cirrhotic portal hypertension, but due to severe thrombocytopenia and a poor general state, we decided not to perform such exam.

A digestive leakage of plasma proteins into the digestive tract had also been ruled out. Clearance of alpha 1‐antitrypsin on a 72 h stool collection was 8,4 ml/24 h and was considered normal.

A bone marrow aspiration was undertaken because of severe thrombocytopenia. The examination showed vacuolated lymphocytes, and megakaryocytes were not viewed. There were no signs of dysplasia.

At the end of this assessment, the suspected cause of the ascites was either portal hypertension or protein leakage from either proteinuria or exudative enteropathy. Literature on ascites in children with sialidosis was scarce and reported a limited life expectancy. Palliative care was provided. Invasive procedures such as kidney/liver biopsy or transjugular intrahepatic portosystemic shunt to reduce portal tension were not realised in this context of poor general state and limited life expectancy.

The management included weekly albumin and platelet transfusions, loop diuretics (furosemide) and aldosterone receptor antagonists (spironolactone). ACE inhibitors (Enalapril) were prescribed to reduce proteinuria. Oxygen administration was necessary to maintain a saturation greater than 94%. He was fed with hydrolysed and enriched milk.

The patient was discharged home and a few weeks later developed multiorgan failure with uncontrollable ascites and respiratory failure which led to death. No autopsy was performed.

## 3 DISCUSSION

We herein report a case of infantile sialidosis diagnosed at the age of 8 months of life with symptoms progressing from the age of 4 months. He presented all the clinical features described in the literature^7^, ^9^: significant psychomotor retardation, major hepatosplenomegaly, enlarged metaphyses, deafness, bilateral inguinal hernia and recurrent respiratory infections. At the age of 2.5 years, his medical condition got worse, developing ascites with minor renal and medullary involvement. To the best of our knowledge, ascites has not yet been described in the infantile form of the disease.

On the contrary, hydrops fetalis and ascites are well known in the congenital form of sialidosis. As for other lysosomal storage disorders ascites probably results from non‐immunological process.^10^, ^25^ The origin of accumulation of excessive fluid within the peritoneal cavity is a source of controversy in the literature.^11^ They progress to stillbirth or death shortly after birth. The mechanism for hydrops fetalis development may involve the obstruction of venous blood flow resulting from visceromegaly secondary to accumulation of storage material.^12^ Sergi et al. described a massive micro‐ and macrovacuolation in the cytoplasm of foetal cells of many organs (liver, bone marrow, kidney, etc.). In the liver, the hepatocytes contained mostly large empty vacuoles, whereas a foamy pattern caused by the presence of vacuoles of variable size was seen in the Kupffer cells. The intraacinar fields showed diffuse extramedullary haematopoiesis. Fibrosis was not seen in the portal tracts.^13^

In this case report, ascites appeared during the evolution of the disease and seems to result from multiple origins: on the one hand, portal hypertension and on the other hypoalbuminemia maintained by proteinuria secondary to nephrosialidosis. The identification of oesophageal varices, petechiae on the gastric mucosa, splenic varices and abdominal venous collateral circulation are suggestive of portal hypertension even if it was not observed at the liver Doppler ultrasound. Analysis of liquid from abdominal paracentesis shows serum‐ascites albumin gradient greater than 1.1 g/dl, further supporting portal hypertension after exclusion of a cardiac involvement.

**FIGURE 1:**
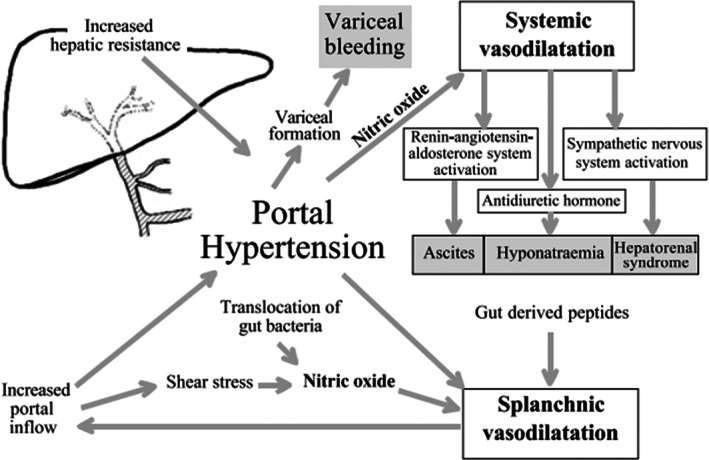
Pathogenesis of portal hypertension

Although portal hypertension has never been described in sialidosis, we can draw a parallel with Gaucher disease because of reticulo‐endothelia cells involvement in both diseases. Gaucher disease is characterised by β‐ Glucocerebrosidase deficiency causing the accumulation of glucocerebroside deposits in the reticuloendothelial system of the liver, spleen and bone marrow. Hepatomegaly is therefore common. Progression to non‐cirrhotic portal hypertension or to fibrosis has been reported in the literature but remain exceptional. The pathophysiology involves infiltration of hepatic sinusoids leading to increased portal pressure^14^, ^15^, ^16^, ^17^ with the attendant complications^18^, ^19^ (see Figure 1).

The *Neu1*−/− knockout (KO) mouse model was helpful in the understanding of underlying molecular mechanisms and standard features of sialidosis. Pathological studies of livers revealed initial ballooning followed by progressive, age‐dependent, filling of sinusoidal cells and hepatocytes with vacuoles.^9^

Although this remains a rare complication in Gaucher disease,^20^, ^21^ loss of plasma proteins in the gastrointestinal tract (protein‐losing enteropathy) in the case of portal hypertension should also be considered. Portal hypertension impairs splanchnic lymphatic drainage causing elevated pressures with dilation of intestinal lymphatic vessels which leads to protein leakage through the intestinal epithelium.^20^, ^22^ Furthermore, Ramaswani's recent publication^23^ focusing on the lesser‐known complications of Gaucher disease reports the development of mesenteric lymphadenopathy and mural thickening of the intestine associated with protein‐losing enteropathy characterised by malnutrition, weight loss and peripheral oedema. Protein‐losing enteropathy was not observed in this case.

The renal involvement in sialidosis often presents as nephrotic syndrome. Vacuoled glomerular cells lead to diffuse mesangial sclerosis, causing proteinuria^1^, ^9^ In nephrotic syndrome, the onset of oedema occurs at serum albumin levels <25 g/L.^24^ Plasma protein of this patient was at the limit level (27 g/L) and the urine protein to creatinine ratio was elevated, but was likely overestimated because of low urinary creatinine for the age and low weight of the child. Proteinuria contributed to maintain a lower albumin level but was probably not sufficient to explain alone the clinical picture.

NEU1 pathogenic mutation (c.599C>T, p.Pro200Leu) has already been described in patients with sialidosis.^8^ A compound heterozygous patient of the c.599C>T mutation and an alternative pathogenic alteration displayed mild type I sialidose, while a c.599C>T homozygous patient showed a severe type II sialidosis.

Alteration c.353‐11A>G, p? has not been described in the literature. However, 4/4 of prediction tools performed by the laboratory of Centre for Medical Genetics (CMG) of the Universitair Ziekenhuis Brussel suggest the creation of a novel splice acceptor site in intron 2, suggesting a disruption in the mature protein. This alteration is classified as variant of uncertain clinical significance (VUS). This alteration has only been detected once in GnomAD at all. This results in an extremely low allele frequency of 0.0004%.

The biochemical markers, the absent enzymatic activity, the genetic results and the phenotype give an evocative picture of sialidosis. The association of these two mutations seems to give a severe phenotype.

In conclusion, this first description of ascites in the infantile form of sialidosis is likely to result from multifactorial origin and is probably suggestive of a rapidly advancing course of disease with poor prognosis. Protein‐losing enteropathy is a rare complication but should be considered in case of hypoalbuminemia. Proteinuria in nephrosialidosis should also be sought in case of hypoalbuminemia.

## FUNDING INFORMATION

The authors received no financial supports for this case report.

## CONFLICT OF INTEREST

The author declares that there is no conflict of interest that could be perceived as prejudicing the impartiality of the research reported.

## ETHICS STATEMENT

An ethics approval statement was not required for this report.

## INFORMED CONSENT STATEMENT

All procedures followed were in accordance with the ethical standards of the responsible committee on human experimentation (institutional and national) and with the Helsinki Declaration of 1975, as revised in 2000. Informed consent was obtained from the parents of the patient for being included in the study. Proof that informed consent was obtained must be available upon request.

## DOCUMENTATION OF APPROVAL FROM THE INSTITUTIONAL COMMITTEE FOR CARE AND USE OF LABORATORY ANIMALS

This article does not contain any animal studies performed by the authors.

## Data Availability

This manuscript has no associated data.
